# A frequency-dependent decoding mechanism for axonal length sensing

**DOI:** 10.3389/fncel.2015.00281

**Published:** 2015-07-21

**Authors:** Paul C. Bressloff, Bhargav R. Karamched

**Affiliations:** Department of Mathematics, University of UtahSalt Lake City, UT, USA

**Keywords:** axonal length control, biochemical oscillations, frequency decoding, gene network, protein thresholds, intrinsic noise

## Abstract

We have recently developed a mathematical model of axonal length sensing in which a system of delay differential equations describe a chemical signaling network. We showed that chemical oscillations emerge due to delayed negative feedback via a Hopf bifurcation, resulting in a frequency that is a monotonically decreasing function of axonal length. In this paper, we explore how frequency-encoding of axonal length can be decoded by a frequency-modulated gene network. If the protein output were thresholded, then this could provide a mechanism for axonal length control. We analyze the robustness of such a mechanism in the presence of intrinsic noise due to finite copy numbers within the gene network.

## 1. Introduction

Size homeostasis is fundamental to cell biology. The ability of a cell to assess its own size or length allows for proper regulation of biochemical processes to meet physiological requirements. Several mechanisms by which cells determine the sizes of subcellular structures have been identified. Some examples include molecular rulers, quantal synthesis, and dynamic balance (Marshall, 2004). The roles that such mechanisms play for relatively small cells in sensing size have been delineated, but it is largely unclear how large cells are able to accomplish this. The problem is particularly acute for neurons, which, in addition to being large cells, exhibit the most variety in cellular size, ranging from a micron to a meter in length in humans.

It is likely that different growth mechanisms act as the underlying length sensors for axons at different stages of development. The initial growth rate of an axon is determined by pre-programmed transcription factor levels (Lallemend and Ernfors, 2012), whereas the interstitial growth rates of axons that have connected to their targets are driven by by the stretching of the organism (Smith, 2009). A central question is whether or not there exists an intrinsic length sensing mechanism in axons that can coordinate between the output of transcriptional and metabolic processes controlled by the nucleus and the differential growth and maintenance needs of axons of different sizes. Theoretical analysis and *in vitro* experimental studies of axonal growth in a variety of neuronal types support the existence of intrinsic length sensors (Goslin and Banker, 1989; Samsonovich and Ascoli, 2006; Brown et al., 2008; O'Toole et al., 2008; Kam et al., 2009), but the underlying mechanisms are largely unknown. Initially, it was hypothesized that molecular diffusion was the fundamental mechanism for sensing length, but given the lengths involved, it is unlikely that diffusion is the underlying mechanism. A more tenable early hypothesis for a length sensor in axons involved the use of molecular rulers. This idea certainly solves the problem of a mechanism accounting for large amounts of growth in relatively small amounts of time; however, the sheer variability in the lengths of axons renders a molecular ruler based length sensing mechanism unfeasible.

**Figure 1:**
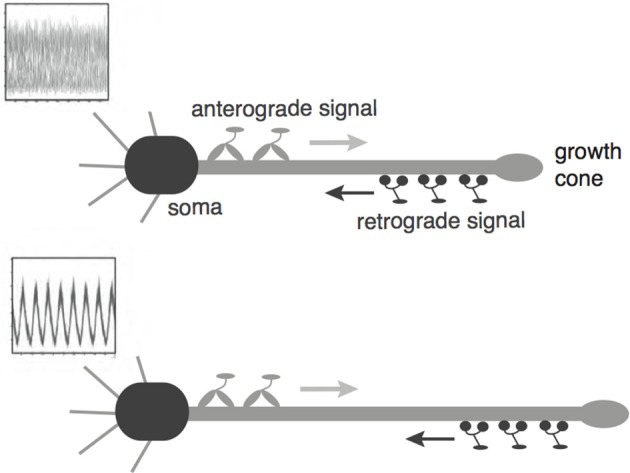
**Schematic diagram of the bidirectional motor-transport mechanism for axonal length sensing hypothesized by Rishal et al. (2012)**. A kinesin-based anterograde signal activates a dynein-based retrograde signal that itself represses the anterograde signal via negative feedback. The frequency of the resulting oscillatory retrograde signal decreases with axonal growth.

Recently, a mechanism for axonal length sensing based on bidirectional motor transport was proposed by Rishal et al. (2012), which is distinct from the aforementioned mechanisms. A schematic illustration of the motor-based model is shown in Figure 1. A signal is produced at the cell body and is subsequently carried by kinesin motors to the tip of the growing axon, where it activates the production of another chemical signal. The latter is transported by dynein motors back to the cell body, where it inhibits the production of the original chemical signal via negative feedback. Once the axon grows past some critical length, the amount of inhibitory chemical signal located at the cell body begins to oscillate with a frequency that decreases as axonal length increases. If axonal growth is correlated with this frequency, then spatial information regarding length of the axon can be communicated to the cell body, where frequency-dependent activation of transcription factors could regulate axonal growth (Cai et al., 2008). Computer simulations of this mechanism provided results that are consistent with what was proposed in Rishal et al. (2012). An important prediction of these simulations is that reducing either anterograde or retrograde signals by partial knockdown of kinesin or dynein motor activity should increase axonal length. This prediction has been confirmed experimentally in peripheral sensory neurons (Rishal et al., 2012). Note that a previous model of Kam et al. (2009) is inconsistent with the experimental data. The earlier model assumes that the unidirectional transport of a retrograde signal by dynein motors maintains axonal growth until the signal at the cell body becomes too weak due to a constant rate of signal loss en route. In this case, the partial knockdown of motor activity would lead to shorter axons. Hence the experimental results provide circumstantial evidence for frequency-encoded axonal length.

Such cellular behavior has been shown to exist in the context of protein production in response to the gonadotropic releasing hormone (GnRH), which pulses at various frequencies over time (Krakauer et al., 2002). Distinct frequencies have been observed to induce the production of disparate proteins. This phenomenon was mathematically analyzed in Krakauer et al. (2002). The results suggest that cellular decoding of frequency-encoded information is possible due to the difference in time scales for gene activity and protein lifetime. Even more interestingly, it has been shown that cells are able to keep protein levels with less variability in response to a pulsatile signal as opposed to a constant signal (Tostevin et al., 2012).

We have recently developed a mathematical version of the computational model given by Rishal et al. (2012), which provides analytical insights into the proposed dynamical mechanism underlying the frequency-encoding of axonal length (Karamched and Bressloff, 2015). The simplest version of the model consists of a pair of delay-differential equations that keeps track of the chemical signals at the somatic and distal ends of the axon. The dynamics of kinesin and dynein motors are not modeled explicitly; instead, their active transport is assumed to introduce a discrete delay that varies linearly with axonal length. We showed how oscillations arise at a critical axonal length via a Hopf bifurcation, and obtained a length-dependent frequency consistent with the previous computational model. In this paper we explore another major aspect of the proposed axonal length-sensing mechanism, namely, how the frequency-based information about axonal length could be decoded at the cell body. As briefly suggested by Rishal et al. (2012), one possibility is that that the frequency-dependent signal could modulate the nuclear import of a transcription factor and thus coordinate the regulation of gene expression. Although the frequency-dependent modulation of gene expression has been observed in yeast (Cai et al., 2008), for example, the role of such a mechanism in axonal length-sensing is currently unknown. Indeed, there is only indirect evidence for the frequency-encoding mechanism itself (Rishal et al., 2012). However, this is precisely a situation where mathematical modeling can play a role, namely, in exploring the consequences of a hypothesized model. Indeed, in our previous work we showed that when the stochastic nature of motor transport is taken into account, there may be a deterioration in the accuracy of the length-encoding mechanism in the case of long axons. In this paper we show that an analogous problem may occur for the length-decoding mechanism when the intrinsic noise of a gene network is included.

In order to develop the basic theory, we feed the oscillating retrograde signal from the delayed feedback model into a simple feedforward gene network. Following along similar lines to Krakauer et al. (2002), we show how even a simple gene network can convert a frequency code to an amplitude code, resulting in a mean protein output that is a monotonically decreasing function of axonal length. If the protein output were thresholded, then this could provide a mechanism for axonal length control, under the assumption that when the protein output falls below threshold, this activates or inactivates another gene circuit that plays a crucial role in axonal growth. Analogous thresholding mechanisms have been investigated within the context of intracellular protein concentration gradients, which are used to determine spatial position within a cell so that, for example, cell division occurs at the appropriate time and location (Tostevin et al., 2007; Tostevin, 2011). Similarly, developmental morphogen gradients control patterns of gene expression so that each stage of cell differentiation occurs at the correct spatial location within an embryo. For biological effectiveness, these gradient-based mechanisms must be robust to intrinsic and extrinsic cellular noise (Tostevin, 2011; Howard, 2012). The main aim of our paper is to demonstrate how the issue of robustness to noise carries over to the proposed frequency decoding mechanism and, hence, that it can be analyzed along similar lines to protein concentration gradients. The structure of the paper is as follows. In Section 2 we briefly review our previous delayed-feedback model for the frequency-based encoding of axonal length and then show how the frequency can be decoded by a simple feedforward gene network. In Section 3 we consider a stochastic version of the gene network and estimate the variance in the protein output concentration along the lines of Tostevin et al. (2012). For completeness, we fill in the details of the calculations whose results were quoted in Tostevin et al. (2012). It should also be noted that these authors focused on comparing the variance in protein output when the input signal is oscillatory with the corresponding variance for constant inputs, establishing that the former is smaller. Here we are interested in the effects of intrinsic noise on frequency decoding itself.

## 2. Model

### 2.1. Delayed feedback model of frequency encoding

We begin by briefly reviewing the delayed feedback model presented in Karamched and Bressloff (2015). Consider an axon of length *L* with *x* = 0 corresponding to the proximal end (adjacent to the cell body or soma) and *x* = *L* corresponding to the distal end (axonal tip). Let *u*_*E*_(*t*) represent the anterograde chemical signal at *x* = *L* at time *t*. This is the excitatory chemical from the mechanism proposed by Rishal et al. (2012), which is produced at the proximal end and carried by kinesin motors to the distal end. Similarly, let *u*_*I*_(*t*) represent the retrograde signal at *x* = 0 at time *t*, which is transported from the distal end by dynein motors. We assume the simplest possible model of active transport, where both types of motors travel at a constant speed *v* along the axon. (Elsewhere we explicitly model the stochastic dynamics of molecular motor transport using a system of advection-diffusion equations, see Karamched and Bressloff, 2015). This assumption means that for a given axonal length *L*, there is a delay τ ≡ *L* ∕ *v* between the production of a chemical signal at one end and its arrival at the opposite end. Finally, exploiting the fact that axonal growth occurs on a much slower time scale than that of motor transport, we treat *L* as fixed and investigate the occurrence of chemical oscillations for a given delay. This then determines a relationship between the frequency of the retrograde signal's oscillation and axonal length. More explicitly, the delayed feedback model takes the form Karamched and Bressloff (2015).

(1) $$\frac{du_{E}}{dt}=I_{0}-\gamma u_{E}-W_{I}f[u_{I}(t-\tau )],$$

(2) $$\frac{du_{I}}{dt}=-\gamma u_{I}+W_{E}f[u_{E}(t-\tau )],$$

with γ decay rate. For simplicity, we take γ to be equal for both chemical signals. The weights *W*_*E*_ and *W*_*I*_ represent the strengths of the positive and negative feedback terms, respectively, based on some form of Michaelis-Menten kinetics. The function *f* can therefore be any monotonically increasing function that saturates to some finite value at infinity. For concreteness, we take *f* to be a Hill function

(3) $$f[u]=\frac{u^{n}}{K^{n}+u^{n}},$$

with dissociation constant *K* and Hill coefficient *n*. The input *I*_0_ represents the constant rate at which the proximal chemical signal is produced in the absence of any negative feedback (*W*_*I*_ = 0). In Karamched and Bressloff (2015) we took *n* = 4 and fixed the scale of the weights *W*_*E*_, *W*_*I*_ and input *I*_0_ by setting *K* = 2. In order to coincide with the results of Rishal et al. (2012) we also set γ^−1^ = 100 sec. Since motor velocities are of order 1 μm/s, it follows that setting τγ = 1 corresponds to an axonal length of 100 μm. (In the following we fix the units of time by setting γ = 1).

**Figure 2:**
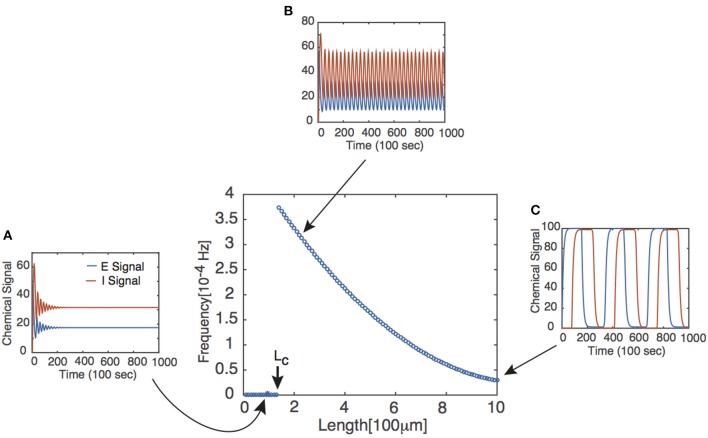
**Frequency of periodic solutions plotted against axonal length**. [Plot was obtained by looking at the power spectrum of the retrograde signal and taking the frequency of the signal to be where the sharp peak of the spectrum occurred.] Insets show time series plots at specific values of the delay generated using the dde23 program in MATLAB: **(A)** τ = 1, **(B)** τ = 2, **(C)** τ = 10. Other parameter values are *n* = 4, *I*_0_ = 10, *W*_*E*_ = *W*_*I*_ = 9.5 such that τ_*c*_ ≈ 1.5.

In Karamched and Bressloff (2015) we carried out a linear stability analysis of Equations (1) and (2) and derived the following Hopf bifurcation conditions (Karamched and Bressloff, 2015):

(4) $$\omega =\cot(\omega \tau ),\;\sqrt{\alpha_{E}\alpha_{I}}\sin(\omega \tau )=1,$$

where ω denotes the frequency of the periodic solution and $\alpha_{P}\equiv W_{P}f^{\prime }\left[u_{P}^{*}\right]$ for *P* = *E*, *I* and $u_{P}^{*}$ the steady state of the corresponding chemical signal. We immediately notice two facts from Equation (4). First, if τ = 0, then the bifurcation conditions cannot be satisfied, suggesting that there exists a critical delay τ_*c*_ past which Equations (1) and (2) have periodic solutions. This corresponds with the existence of some critical axonal length *L*_*c*_ past which signals will oscillate. Second, the bifurcation conditions can only be satisfied if $\sqrt{\alpha_{E}\alpha_{I}}>1$. It follows that the feedback strengths *W*_*P*_ must be sufficiently large and/or the Hill function must be sufficiently steep. The latter implies that oscillations are facilitated if the chemical signal interactions are cooperative in nature, as reflected by the value of *n* in the delayed feedback model. The existence of the Hopf bifurcation point does not in itself guarantee the onset of stable limit cycles for τ > τ_*c*_. However, this can be verified numerically, and one finds that the frequency of the oscillation beyond the bifurcation point is a monotonically decreasing function of *L*, see Figure 2.

In conclusion, the above delayed feedback model makes explicit the role of negative feedback in the frequency encoding mechanism for axonal length sensing. (The same basic mechanism also holds for our more detailed advection-diffusion model, Karamched and Bressloff, 2015). We now turn to a possible mechanism for decoding the frequency of the oscillatory retrograde signal, and thus recovering the axonal length. To that end, we will feed the retrograde signal from our model into a simple gene network with a feedforward network topology. Note that from this perspective, the details of the particular mechanism for generating the oscillatory signal are not important, so we will focus on the simple delayed feedback model given by Equations (1) and (2).

### 2.2. Frequency decoding by a feedforward gene network

Suppose that the oscillating retrograde signal from the delayed feedback model triggers a signaling cascade via the action of certain kinases that ultimately leads to the translocation of a transcription factor into the nucleus of a given neuron and causes the rapid activation of some gene and subsequent production of some protein *C*

$$\begin{array}{l} u_{I}(t)\to \text{phosphorylation and translocation}\\ \;\overset{fast}{\to }\text{Active Gene}\overset{fast}{\to }C\overset{\lambda }{\to }\emptyset \end{array}$$

with λ decay rate. This motivates the following model for the dynamics of protein *C* (Krakauer et al., 2002):

(5) $$\frac{dc}{dt}=h[u_{I}(t)]-\lambda c,$$

where *c* denotes the concentration of protein *C* and *h*[*u*] is a monotonically increasing function satisfying *h* → h^*^ ∈ (0, ∞) as *u* → ∞. This is introduced to reflect the fact that the retrograde signal does not directly activate the gene. Define *g*(*t*) ≡ *h*[*u*(*t*)]. Then *g* is *T*-periodic, where *T* is the period of *u*_*I*_(*t*). Following Krakauer et al. (2002), we obtain the time-dependent solution for *c*(*t*) and show that this simple feedforward network can act as a frequency decoder. Introduce the integrating factor *e*^λ*t*^. Then,

$$\begin{array}{l} \frac{d}{dt}(e^{\lambda t}c(t))=g(t)e^{\lambda t}\\ \;\Rightarrow c(t)=c(t_{0})e^{-\lambda (t-t_{0})}+\int_{t_{0}}^{t}g(s)e^{-\lambda (t-s)}ds. \end{array}$$

We integrate over a period of *u*_*I*_(*t*) so that, for *m* ∈ ℕ,

(6) $$\begin{array}{l} c((m+1)T)=c(mT)e^{-\lambda T}+\int_{mT}^{(m\;+\;1)T}g(s)e^{-\lambda ((m+1)T-s)}ds\\ \;=c(mT)e^{-\lambda T}+e^{-\lambda T}\int_{0}^{T}g(s)e^{\lambda s}ds. \end{array}$$

Equation (6) gives a recursive finite difference equation for *c* at integer multiples of the period of *u*_*I*_(*t*). For large *m*, we thus have

(7) $$c(mT)=\frac{e^{-\lambda T}}{1-e^{-\lambda T}}\int_{0}^{T}g(s)e^{\lambda s}ds.$$

**Figure 3:**
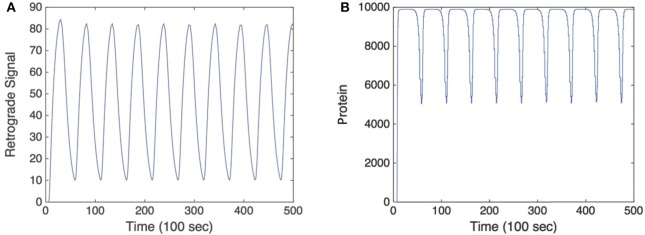
**Simulation of the feed forward serial network Equation (5) in response to a retrograde signal from Equation (2)**. **(A)** Retrograde signal being fed into gene network, τ = 5. **(B)** Convergence of the solutions of Equation (5) to a *T*-periodic solution post transience. *h*[*u*] is taken to be the same function as *f*[*u*] defined in Equation (3) multiplied by a factor of 1000, and we set λ = 0.01. Other parameter values are as in Figure 2.

Hence, *c*(*t*) converges to a *T*-periodic solution following any transient dynamics, as shown in Figure 3. More significantly, there is now a strong DC component to the signal so that the relative amplitude of the oscillatory part has been suppressed. Indeed it is possible to find parameter values for which $c\left(t\right)\approx \bar{c}$, where $\bar{c}$ is the time-averaged protein output (Krakauer et al., 2002). Therefore, in order to characterize the protein output in terms of the frequency ω of *u*_*I*_(*t*), we find the time average of *c*(*t*) post transience. This can be done by simply integrating Equation (5) over a period of *u*_*I*_(*t*):

(8) $$\bar{c}=\frac{1}{\lambda T}\int_{0}^{T}g(s)ds\equiv \frac{\bar{g}}{\lambda }.$$

Equation (8) is an intuitive result. It says that the average protein output from the feedforward serial network is equal to the ratio of the average protein activation rate to the protein decay rate.

To make the relationship between $\bar{c}$ and *T* more explicit, we perform the following. Assume that in the post-transient time regime, the maximum value of *u*_*I*_(*t*) is given by *U*_*M*_ and that the minimum value is given by *U*_*m*_, and that the *u*_*I*_ transitions from *U*_*M*_ to *U*_*m*_ occur very quickly compared to other temporal dynamics. Further assume that *h*[*u*] is a Hill function with a large Hill coefficient, so that *h*[*U*_*M*_] = *A* and that *h*[*U*_*m*_] ≈ 0. Let η < *T* denote the amount of time for which *u*_*I*_(*t*) is at its maximum value in a given period, η = κ*T* for 0 < κ < 1. Then, *ḡ* ≈ *A*η ∕ *T* and

(9) $$\bar{c}\approx \frac{A\eta }{\lambda T}.$$

Note that the assumptions made regarding *u*_*I*_(*t*) are consistent with the behavior of the retrograde signal for sufficiently long delays (see Figure 2).

**Figure 4:**
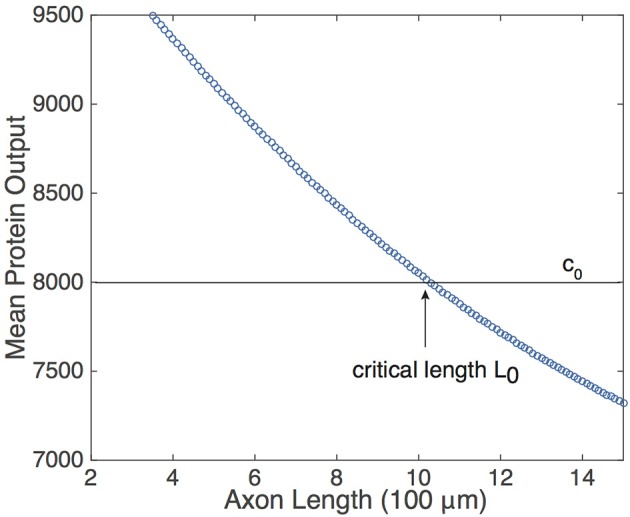
**Relationship of the mean protein output $\bar{c}$ and axonal length *L*, obtained by time averaging the solution to Equation (5) for several values of τ**. Function definitions and parameter values are as in Figures 2, 4. The existence of a threshold protein output *c*_0_ could provide a mechanism for determining a critical length *L*_0_.

Equation (9) suggests that if the protein decay rate λ, the rate of protein activation *A*, and the pulse-width η are constant, then the mean protein output $\bar{c}$ is a monotonically decreasing function of the period *T* of the pulsatile retrograde signal. In the context of the delayed feedback model, this means that $\bar{c}$ is a monotonically decreasing function of axonal length *L*. Although the analytical representation of $\bar{c}$ was obtained by making assumptions that simplified the analysis of Equation (5), and the pulse-width η is not fixed (see Figure 2), one still finds numerically that $\bar{c}$ decreases monotonically with *L*, see Figure 4. (As shown by Krakauer et al. (2002), it is also possible to modify the simple gene network so that the protein output becomes independent of pulse width). Note that if *U*_*M*_ is sufficiently large, then *A* ≈ h^*^ due to the saturating nature of *h*. What is more, changing the value of *U*_*M*_ will not alter $\bar{c}$ significantly unless it is reduced by a considerable amount. Thus, the mean protein output of the system is relatively insensitive to the amplitude of the input signal and responds only to the frequency of the input signal, making the feed forward serial network a plausible means by which a neuron can decode the oscillating retrograde signal from the delayed feedback model.

The monotonic relationship between $\bar{c}$ and *L* suggests that the underlying intrinsic axonal length sensor could be based on a threshold protein value. That is, suppose that a given neuron is pre-programmed to grow until the mean protein output reaches some threshold value, *c*_0_, see Figure 4. Based on the mean protein output, the neuron would be able to sense its critical length *L*_0_ and stop growing, for example. Analogous thresholding mechanisms have been investigated within the context of intracellular protein concentration gradients, which are used to determine spatial position within a cell so that, for example, cell division occurs at the appropriate time and location (Tostevin et al., 2007; Tostevin, 2011). Similarly, developmental morphogen gradients control patterns of gene expression so that each stage of cell differentiation occurs at the correct spatial location within an embryo. For biological effectiveness, these gradient-based mechanisms must be robust to intrinsic and extrinsic cellular noise (Tostevin, 2011; Howard, 2012). The issue of robustness to noise carries over to the proposed axonal-length sensing mechanism, and can be analyzed along similar lines to protein concentration gradients. Therefore, we now investigate the impact of intrinsic noise in a gene network arising from finite copy numbers on the shape of the deterministic $\bar{c}$ vs. *L* curve.

## 3. Effects of intrinsic noise on axonal length sensing

### 3.1. Stochastic gene network

**Figure 5:**
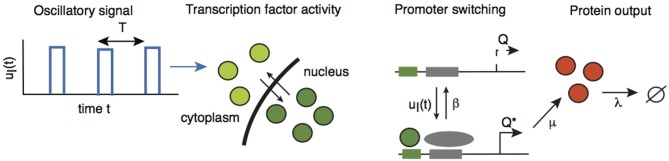
**A gene promoter driven by the oscillatory retrograde signal *u*_*I*_(*t*)**. Adapted and redrawn from Tostevin et al. (2012).

In order to investigate the effects of intrinsic noise, we consider an extended version of the network analyzed in Section 2.2 in which we explicitly include the dynamics of gene activation. Suppose that a gene promoter has two states: an inactive state *Q* and an active state Q^*^. In the active state, the gene produces the protein *C* at a rate of μ, and the protein subsequently decays at a rate λ. The promoter is activated in response to the pulsatile retrograde signal *u*_*I*_(*t*) and deactivates at a constant rate of β, see Figure 5:

(10) $$Q\;\overset{u_{I}(t)}{\underset{\beta }{⇌}}Q^{*}\overset{\mu }{\to }C\overset{\lambda }{\to }\emptyset .$$

Suppose there are *N* total gene promoters, each of which can exist in an active state or an inactive state. If *N* is sufficiently large, then the effects of intrinsic noise are negligible and one can represent the deterministic dynamics using kinetic equations. Let *x*(*t*) and *c*(*t*) denote, respectively, the fraction of active genes and the concentration of proteins (number of proteins per gene) at time *t*. Then

(11) $$\frac{dx}{dt}=s(t)(1-x)-\beta x,\;\frac{dc}{dt}=\mu x(t)-\lambda c(t),$$

where *s*(*t*) is the input to the gene network, which is taken to be the concentration of promoters. The latter is itself controlled by the oscillatory retrograde signal *u*_*I*_(*t*) coming from the delayed feedback model. As in the previous model, we will take *s*(*t*) = *h*[*u*_*I*_(*t*)]. Assume without loss of generality that *x*(0) = 0, so that the solution for *x*(*t*) takes the form

(12) $$x(t)=\int_{0}^{t}s(z)\text{exp}(\beta (z-t)+\int_{t}^{z}s(\xi )d\xi )dz.$$

We would like to calculate the time-averaged level of active genes in the large-time limit. In order to simplify our calculations, we proceed as in Section 2.2 and take the oscillatory signal *s*(*t*) to consist of square pulses of unit height, width η and period *T*. Setting *t* = *MT*, positive integer *M*, we can break up the integrals on the right-hand side of Equation (12) into a sum of integrals evaluated over a single period:

$$\begin{array}{l} x(MT)=\sum_{n\;=\;0}^{M\;-\;1}\int_{nT}^{(n+1)T}s(z)\text{exp}(\beta (z-MT)+\int_{MT}^{z}s(\xi )d\xi )dz\\ \;=\frac{1}{\beta +1}[\text{exp}((\beta +1)\eta )\text{​​}-1]\text{ ​​}\left(\frac{\text{exp}(-\beta MT-M\eta )-1)}{1-\text{exp}(\beta T+\eta )}\right). \end{array}$$

The second line comes from evaluating the various integrals and summing the resulting geometric series. Taking the limit *M* → ∞ shows that *x*(*MT*) → Γ with

$$\Gamma \equiv \frac{1}{\beta +1}[1-\text{exp}((\beta +1)\eta )]\frac{1}{1-\text{exp}(\beta T+\eta )}.$$

For *t* ∈ [0, η], we have *x*(*t*) = *x*_0_(*t*) with

$$\frac{dx_{0}}{dt}=1-(1+\beta )x_{0}\Rightarrow x_{0}(t)=\frac{1}{1+\beta }(1-e^{-(\beta +1)t})+\Gamma e^{-(\beta +1)t},$$

whereas for *t* ∈ (η, *T*], we have *x*(*t*) = *x*_1_(*t*) with

$$\frac{dx_{1}}{dt}=-\beta x_{1}\Rightarrow x_{1}(t)=x_{0}(\eta )e^{-\beta (t-\eta )}.$$

We have imposed continuity of the solution at *t* = η. Finally, $\bar{x}$ is obtained by averaging the resulting periodic function over [0, *T*].

(13) $$\begin{array}{l} \bar{x}=\frac{1}{T}[\int_{0}^{\eta }x_{0}dt+\int_{\eta }^{T}x_{1}dt]\\ \;=\frac{1}{\beta +1}[\frac{\eta }{T}+\frac{1}{T\beta (\beta +1)}\frac{1-e^{\beta (T-\eta )}}{1-e^{\beta T+\eta }}(e^{\eta (\beta +1)}-1)]. \end{array}$$

The formula for $\bar{x}$ given above can be intuited in the following way. The fraction η∕*T* corresponds to the fraction of time that *s*(*t*) is “on”. The latter term in the bracketed sum is a correction for the alterations in the time-scale of the gene promoter reaction to *s*(*t*). When *s*(*t*) = 1, the time-scale of the gene promoter response is given by (β+1)^−1^, whereas when *s*(*t*) = 0, the time-scale is given by β^−1^. Finally, the time-averaged protein output is

$$\bar{c}=\frac{\mu }{\lambda }\bar{x}.$$

As in the simpler gene network of Section 2.2, we find that the time-averaged protein output is a monotonically decreasing function of *T* even though the pulse-width η also changes with *T* as in Figure 2.

Now suppose that *N* is sufficiently small so that fluctuations due to low copy numbers cannot be ignored. In order to calculate the size of fluctuations, we have to consider the chemical master equation of the reaction scheme (Equation 10). Let *n*_1_ denote the total number of activated genes and let *n*_2_ denote the number of proteins that are present. Let *P* ≡ *P*(*n*_1_, *n*_2_, *t*) denote the probability that at a given time *t* there are *n*_1_ active genes and *n*_2_ proteins available. The master equation is then given by

(14) $$\begin{array}{l} \frac{dP}{dt}=s(t)(N-n_{1}+1)P(n_{1}-1,n_{2},t)\\ \;+\beta (n_{1}+1)P(n_{1}+1,n_{2},t)\\ \;+\mu n_{1}P(n_{1},n_{2}-1,t)+\lambda (n_{2}+1)P(n_{1},n_{2}+1,t)\\ \;-(s(t)(N-n_{1})+\beta n_{1}+\mu n_{1}+\lambda n_{2})P(n_{1},n_{2},t). \end{array}$$

The first two terms correspond to the activation or the deactivation of a gene that results in having *n*_1_ active genes and *n*_2_ proteins. The second two terms correspond to the production or the degradation of a protein that results in having *n*_1_ active genes and *n*_2_ proteins. The last terms correspond to the ways that the system can leave the state of having *n*_1_ active genes and *n*_2_ proteins. It is difficult to solve the master equation explicitly, so we carry out a system size expansion with respect to *N*. That is, set *n*_1_ = *Nx*, *n*_2_ = *Nc* and rewrite Equation (14) as

$$\begin{array}{l} \frac{dP}{dt}=N[s(t)\left(1-x+\frac{1}{N}\right)P(n_{1}-1,n_{2},t)\\ \;+\beta \left(x+\frac{1}{N}\right)P(n_{1}+1,n_{2},t)\\ \;+\mu xP(n_{1},n_{2}-1,t)+\lambda \left(c+\frac{1}{N}\right)P(n_{1},n_{2}+1,t)\\ \;-(s(t)(1-x)+\beta x+\mu c+\lambda c)P(n_{1},n_{2},t)]. \end{array}$$

The master equation is now just a sum of terms of the form *f*(**n** ∕ *N*)*P*(**n**, *t*), where **n** ≡ (*n*_1_, *n*_2_) and *f* is the corresponding propensity function. Performing the change of variables *f*(**n** ∕ *N*)*P*(**n**, *t*) = *f*(**x**)*p*(**x**, *t*), where **x** ≡ (*x*, *c*), and Taylor expanding in powers of *N*^−1^ to second order leads to the Fokker-Planck equation

$$\begin{array}{l} \frac{dp}{dt}=-\frac{\partial }{\partial x}([s(t)(1-x)-\beta x]p)-\frac{\partial }{\partial c}([\mu x-\lambda c]p)\\ \;+\frac{1}{2N}\frac{\partial^{2}}{\partial x^{2}}([s(t)(1-x)+\beta x]p)+\frac{1}{2N}\frac{\partial^{2}}{\partial c^{2}}([\mu x+\lambda c]p). \end{array}$$

In the case of a constant input *s*(*t*) = α, the deterministic kinetic Equation (11) have the unique fixed point solution

$$x^{*}=\frac{\alpha }{\alpha +\beta },\;c^{*}=\frac{\mu }{\lambda }x^{*}.$$

In this case, neglecting transients, the Fokker-Planck equation describes a stochastic process characterized by Gaussian fluctuation about the fixed point (x^*^, c^*^). It is then relatively straightforward to calculate the stationary variance Δ*c* of the protein output, given the mean ⟨*c* ⟩ = c^*^:

$$\begin{array}{l} \Delta c=\frac{1}{\gamma }[\frac{\mu^{2}\alpha \beta }{(\alpha +\beta +\lambda ){\left(\alpha +\beta \right)}^{2}}+\frac{\alpha \mu }{\alpha +\beta }]\\ \;=\frac{\mu }{\lambda }\frac{\alpha }{\alpha +\beta }[1+\frac{\mu \beta }{\left(\alpha +\beta +\lambda )(\alpha +\beta \right)}]\\ \;=\langle c\rangle [1+\frac{\mu \beta }{\left(\alpha +\beta +\lambda )(\alpha +\beta \right)}]. \end{array}$$

The expression for the variance in the case of constant input consists of an intrinsic Poissonian term due to random protein production and an extrinsic term due to fluctuations in the gene promoters themselves. The calculation of the variance in the case of an oscillatory input *s*(*t*) is considerably more involved, even when it takes the form of square pulses. However, stochastic simulations show that the protein variance in response to an oscillatory signal is less than the protein variance in response to a constant input, assuming that time-averaged means are the same (Tostevin et al., 2012). Let α_eff_ be the effective constant input for which the time-averaged and noise-averaged protein output $\left\langle \bar{c}\right\rangle$ can be written as

(15) $$\langle \bar{c}\rangle =\frac{\mu }{\lambda }\frac{\alpha_{\text{eff}}}{\alpha_{\text{eff}}+\beta }.$$

It follows that for an oscillatory input

(16) $$\Delta \bar{c}\leq \langle \bar{c}\rangle [1+\frac{\mu \beta }{\left(\alpha_{\text{eff}}+\beta +\lambda )(\alpha_{\text{eff}}+\beta \right)}],$$

where

$$\Delta \bar{c}(L)=\langle {\left[\bar{c}(L)-\langle \bar{c}(L)\rangle \right]}^{2}\rangle .$$

**Figure 6:**
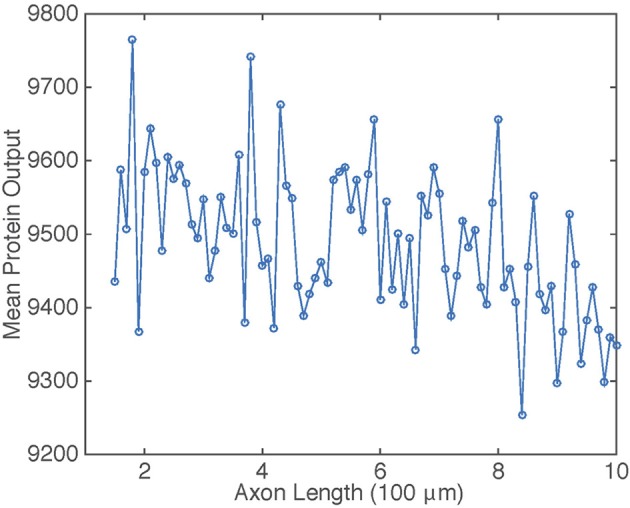
**Plot of mean protein output $\bar{c}$ vs. axonal length *L***. Results are based on simulations of the chemical master Equation (14) using the Gillespie algorithm with input *s*(*t*) = *h*[*u*_*I*_(*t*)]. Parameter values used to generate retrograde signal *u*_*I*_(*t*) are the same as in Figure 2. Other parameter values are β = 1, μ = 0.1, λ = 0.01, and *N* = 1000.

The dependence of $\bar{c}$ on axonal length *L* in the presence of intrinsic noise is shown in Figure 6. The general inverse relationship is still prevalent in this situation, but fluctuates due to the stochasticity in the gene switching.

### 3.2. Errors in axonal length-sensing

**Figure 7:**
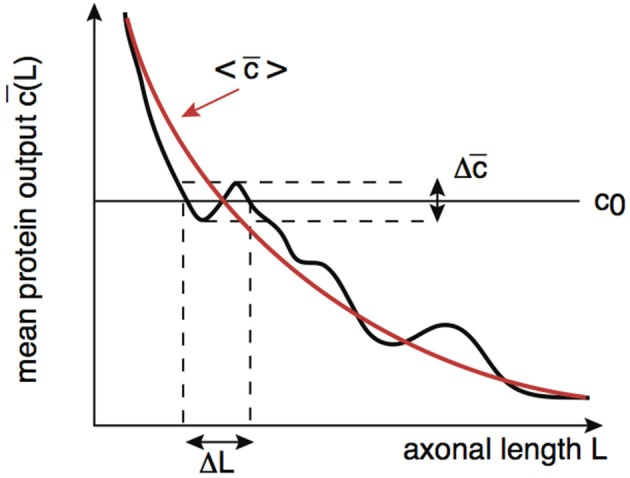
**Schematic diagram illustrating how the presence of noise in the protein output leads to an uncertainty Δ*L* in the critical axonal length *L*_0_ at which the threshold is crossed**. An analogous result applies to decoding of protein concentration gradients.

By analogy with the effects of intrinsic noise in protein concentration gradients (Howard, 2012), the presence of noise in the protein output leads to an uncertainty Δ*L* in the critical axonal length *L*_0_ at which the threshold *c*_0_ is crossed. This is illustrated schematically in Figure 7. Although the time-averaged protein output $\bar{c}$ is still approximately a monotonically decreasing function of axonal length *L* on large length-scales, fluctuations due to intrinsic noise mean that it is non-monotonic on smaller length scales. It follows that the protein output can cross threshold several times over small changes in *L* resulting in an uncertainty Δ*L* with regards to the critical axonal length. The uncertainty Δ*L* can be estimated as follows: Suppose that in the absence of noise $\bar{c}\left(L_{0}\right)=c_{0}$. Denoting the variance in the concentration due to intrinsic noise by $\Delta \bar{c}\left(L_{0}\right)$, we then have the approximation

$$\begin{array}{l} c_{0}=\bar{c}(L_{0}+\Delta L/2)+\frac{1}{2}\sqrt{\Delta \bar{c}(L_{0})}\approx \bar{c}(L_{0})-\frac{\Delta L}{2}|{\bar{c}}^{\prime }(L_{0})|\\ \;+\frac{1}{2}\sqrt{\Delta \bar{c}(L_{0})}, \end{array}$$

which yields the result

(17) $$\Delta L|\langle {\bar{c}}^{\prime }(L_{0})\rangle |=\sqrt{\Delta \bar{c}(L_{0}))}.$$

If we ignore the correction factor in Equation (16) and approximate the stochastic process by a Poisson process, then $\Delta \bar{c}\approx \bar{c}$ and

$$\Delta L\sim \frac{\sqrt{\langle \bar{c}(L_{0})\rangle }}{\left|\langle {\bar{c}}^{\prime }(L_{0})\rangle \right|}.$$

**Figure 8:**
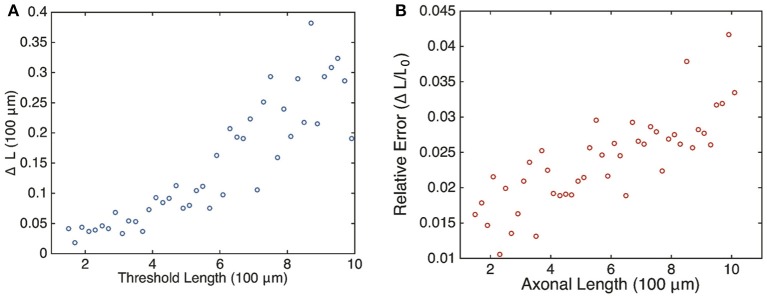
**Plot of estimated errors in axonal length based on 100 simulations of the chemical master Equation (14) using the Gillespie algorithm with input *s*(*t*) = *h*[*u*_*I*_(*t*)]**. **(A)** Plot of uncertainty in axonal length Δ*L* vs. threshold axonal lengths *L*_0_. **(B)** Relative error (Δ*L* ∕ *L*_0_) vs. axonal length. Same parameters as Figure 6. *L*_0_ was found by averaging over the mean protein outputs and determining what length that protein value corresponded to according to the curve shown in Figure 4. Δ*L* was determined by looking at what axonal length each individual mean protein output realization corresponded to according to Equation (4) and then finding the variance in this set of values.

As a further approximation, suppose that $\left\langle \bar{c}\left(L\right)\right\rangle \sim 1∕T$, where *T* is the period of oscillations produced by an axon of length *L*, so that $|\left\langle {\bar{c}}^{\prime }\left(L\right)\right\rangle |\sim 1∕\left(L^{\prime }\left(T\right)T^{2}\right)$ (using the fact that *L* increases monotonically with *T* and *L*(*T*) is the inverse of the function *T*(*L*)). It follows that

$$\frac{\Delta L}{L_{0}}\sim \frac{T_{0}^{3/2}L^{\prime }(T_{0})}{L(T_{0})},$$

with *T*_0_ the oscillation period at the critical length *L*_0_, i.e., *L*(*T*_0_) = *L*_0_. Assuming that the length *L* increases at least linearly with *T*, we see that the relative error grows with the critical oscillation period *T*_0_ and, hence, the critical axonal length *L*_0_. Although this is a crude estimate, we find that the same qualitative behavior is observed in numerical simulations of the full stochastic model. This is shown in Figure 8, where we plot the relative error Δ*L* ∕ *L*_0_ vs. axonal length. Our analysis suggests that the frequency-encoded protein threshold mechanism could break down for long axons. An analogous result was shown to hold in Karamched and Bressloff (2015), where the robustness of the encoding of axonal length in the frequency of a pulsatile signal was investigated. There we found that the encoding of axonal length into frequency became less reliable at long axon lengths due to accumulation of white noise signified by a high coefficient of variation in the frequency of the retrograde signal. In this work, the retrograde signal is deterministic, and the error in protein output is accounted for strictly by the random variations in the activities of independent gene promoters. Hence the error in length sensing could be more devastating in real life situations, since noise would impact both the encoding and the decoding processes. Thus, wherever the sources of noise may be, their impact on this frequency-dependent mechanism is clear: large neurons would have a more difficult time sensing their own length when compared with smaller neurons.

## 4. Discussion

In this paper we extended the recent delayed-feedback model of axonal length-sensing based on the frequency of an oscillating retrograde signal (Rishal et al., 2012; Karamched and Bressloff, 2015) in order to investigate the issue of frequency decoding. In particular, we showed that the mean protein output of a simple feedforward gene network responding to the pulsatile retrograde signal of the delayed feedback model varies inversely with axonal length. Specifically, we derived approximate analytical results which make explicit the inverse relationship, and introduced the notion that frequency decoding could be done based on a protein threshold mechanism. We then investigated the reliability of such a mechanism subject to intrinsic noise stemming from finite copy numbers within a gene network by analyzing a chemical master equation, which describes the random switching of genes and production of protein. The results of these simulations suggest that the accuracy in the information the neuron receives regarding axonal length declines as axonal length itself grows. The latter could have serious implications for the utility of this mechanism in the context of axonal injury, where accurate information regarding the locality of an affliction is necessary for a neuron to set a regenerative process in motion. Of course, the noise sensitivity could be a consequence of using a simple feedforward gene network. Our work shows that there needs to be some additional processing to increase the robustness to noise, perhaps by including some form of feedback. Indeed, one can view our simple gene network as the first stage in a more complex network that carries out the thresholding of the protein output. For example, one could consider a mutual repressor model, which consists of two repressor proteins whose transcription is mutually regulated, that is, the protein product of one gene binds to the promoter of the other gene and represses its output (Gardner et al., 2000). Such a network can act as a bistable switch. If one of the genes were also driven by the retrograde signal as outlined in Section 3, then the switch could be activated or inactivated below a critical frequency. We hope to explore the effects of noise in this more complex network elsewhere—here we wanted to separate out the frequency decoding mechanism from the thresholding mechanism.

There are several other issues we hope to explore in the future. First, we would like to feed the retrograde signal arising from a model that more accurately describes motor dynamics into the feedforward serial gene network. For example, an advection-diffusion equation could be used to model motor dynamics (Karamched and Bressloff, 2015), and the aforementioned chemical signal network could be coupled to these dynamics to generate a pulsatile retrograde signal. It would be interesting to take this idea to a higher level and perform a fully stochastic simulation of motor dynamics, where we allow for binding and unbinding of motors to microtubular tracks. Such a simulation would allow us to characterize noise in the system better, and provide more accurate representations of errors inherent in the axon length sensing mechanism. Finally, Equation (9) indicates that mean protein output is sensitive to the frequency of the incoming pulse signal, but that it is also sensitive to the fraction of time for which the incoming signal is at its peak value. Hence the feedforward serial network does not generate a frequency filter in the strictest sense. We would be interested in seeing the result of feeding the retrograde signal into a network that allows for more acute frequency sensitivity.

Finally, we note that an alternative axonal length-sensing mechanism has been proposed by Roossien et al. (2013). These authors studied axonal growth in Drosophila neurons. In particular, they tracked the movement of docked mitochondria in order to establish that the physical mechanism of growth cone advance in Drosophila is similar to vertebrate neurons. That is, the bulk forward translocation of microtubules along the axon underlies the advance of the growth cone C-domain. They also compared the length of axons grown on two different substrates, either poly-ornithine or Drosophila ExtraCellular Matrix (DECM). They found that axons grown on the faster substrate DECM ended up being longer than the other substrate. The authors suggested that if a length sensor were the sole regulator of the cessation of elongation, then neurons grown on poly-ornithine would be expected to sustain elongation for a longer time than neurons grown on DECM so that they end up having similar lengths. Since this was not observed, it suggests that there may be some internal clock that is independent of axonal length and terminates elongation after a set period of growth.

## Author contributions

PB designed the research; PB and BK developed the analysis; BK performed the numerical simulations; PB and BK wrote the paper.

### Conflict of interest statement

The authors declare that the research was conducted in the absence of any commercial or financial relationships that could be construed as a potential conflict of interest.
